# Performance of DOAC and HAS-BLED scores in predicting major bleeding in Asian patients with non-valvular atrial fibrillation receiving direct oral anticoagulants

**DOI:** 10.1093/europace/euaf251

**Published:** 2025-10-03

**Authors:** Yi-Hsin Chan, Yi-Wei Kao, Shao-Wei Chen, Tze-Fan Chao

**Affiliations:** The Cardiovascular Department, Chang Gung Memorial Hospital, Linkou, Taoyuan, Taiwan; College of Medicine, Chang Gung University, Taoyuan, Taiwan; School of Traditional Chinese Medicine, College of Medicine, Chang-Gung University, Taoyuan City, Taiwan; Microscopy Core Laboratory, Chang Gung Memorial Hospital, Linkou, Taoyuan, Taiwan; Department of Applied Statistics and Information Science, Ming Chuan University, Taoyuan City, Taiwan; Artificial Intelligence Development Center, Fu Jen Catholic University, Taipei, Taiwan; Division of Thoracic and Cardiovascular Surgery, Department of Surgery, Linkou Medical Center, Chang Gung Memorial Hospital, Chang Gung University, Taoyuan City, Taiwan; Center for Big Data Analytics and Statistics, Chang Gung Memorial Hospital, Taoyuan, Taiwan; Division of Cardiology, Department of Medicine, Taipei Veterans General Hospital, No. 201, Sec. 2, Shih-Pai Road, 112062,Taipei, Taiwan; Institute of Clinical Medicine, Cardiovascular Research Center, National Yang Ming Chiao Tung University, 11221, Taipei, Taiwan

**Keywords:** Bleeding risk assessment, DOAC, HAS-BLED, Non-valvular atrial fibrillation

## Abstract

**Aims:**

The direct oral anticoagulant (DOAC) score was recently developed to predict bleeding risk in patients with atrial fibrillation (AF) receiving oral anticoagulants. However, limited data show inconsistent results comparing its performance to the conventional HAS-BLED score in Asian populations with non-valvular AF receiving DOACs.

**Methods and results:**

We enrolled 21 142 patients with non-valvular AF receiving DOACs from a multicentre database in Taiwan (June 2012–December 2021). The primary endpoint was major bleeding events. Major bleeding events were defined according to the ISTH criteria. Areas under receiver operating characteristic curves (AUCs) were calculated for each score, with differences assessed using DeLong test. A total of 21 142 AF patients (mean age 75.9 ± 11.0 years; 41% female) treated with DOAC were included in the analysis. Major bleeding events occurred in 681 patients in 1-year follow-up (3.66%/year). There were 82(0.43%/year) intracranial haemorrhage event occurred. Both the DOAC and HAS-BLED scores are associated with a significant risk of major bleeding event, with only modest predictive performance (AUC < 0.7). The DOAC score showed a slightly but statistically significantly higher AUC compared with the HAS-BLED score [AUC: 0.670 (95% CI: 0.650–0.689) vs. 0.642 (0.623–0.663); *P* < 0.001]. Results from several reclassification analyses favoured the DOAC score. Both the two scores showed a good calibration for the low to intermediate risk categories, while the two bleeding risk scores both overestimate the risk of major bleeding risk for the high risk categories. Subgroup analyses indicated that the superiority of DOAC score over HAS-BLED score is primarily driven by elderly patients (≥75 years) and prediction in risk of gastrointestinal bleeding.

**Conclusion:**

The DOAC score, which employs a more granular scoring system compared to the HAS-BLED score, may enable finer bleeding risk discrimination among Asian patients with non-valvular AF receiving DOAC therapy.

## Graphical Abstract

**Graphical Abstract:**
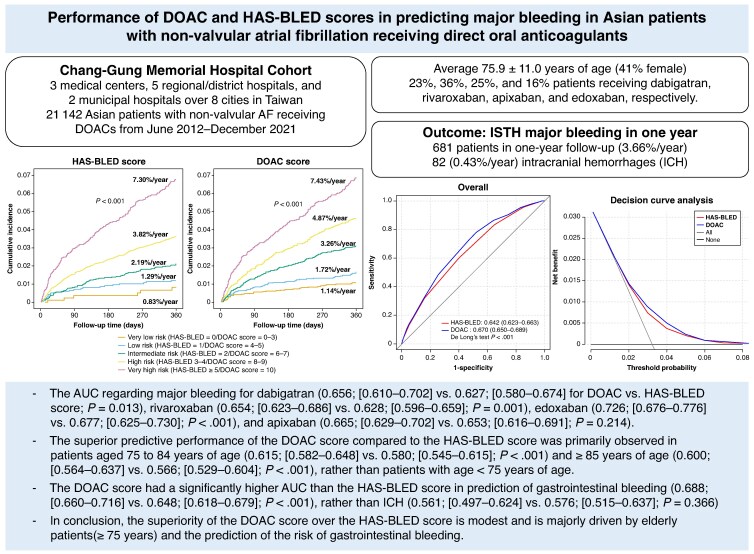

What’s new?

The direct oral anticoagulant (DOAC) score provides a valuable, tailored bleeding risk assessment tool for Asian patients with non-valvular atrial fibrillation receiving DOAC therapy, thereby enhancing clinical decision-making and facilitating shared discussions between clinicians and patients. The superiority of the DOAC score over the HAS-BLED score is modest and is majorly driven by elderly patients (≥75 years) and the prediction of the risk of gastrointestinal bleeding.

## Introduction

Atrial fibrillation (AF) is the most common cardiac arrhythmia worldwide,^1^ and it is associated with a five-fold increased risk of stroke and a two-fold increased risk of mortality when compared to patients without AF.^2^ Direct oral anticoagulants (DOACs) are an effective, safer, and more convenient alternative to warfarin in patients with non-valvular AF, even in the presence of multiple frailty conditions such as advanced age, low body weight, chronic kidney or liver disease, or malignancy.^2–7^ The observed low overall mortality from stroke and bleeding in contemporary AF registries reflects the substantial improvements in AF management, particularly the widespread adoption of DOACs in the real-world practice.^8–10^ Accurate bleeding risk assessment is crucial for optimizing patient care and outcomes in AF patients taking oral anticoagulants (OACs), with major bleeding accounting for roughly 10% of mortalities.^8,9^ Several bleeding risk assessment scores are used to evaluate bleeding risk in AF patients receiving DOACs.^11–14^ The HAS-BLED score is the most commonly used due to its simplicity and validation in multiple populations,^15^ incorporating factors such as uncontrolled hypertension, abnormal renal or liver function, stroke history, bleeding history, labile INR (for warfarin users), elderly > 65 years of age, and drug or alcohol use, with a score of ≥3 indicating high risk.^11^ The use of HAS-BLED is endorsed by major AF guidelines focused on Asian populations,^3,6^ while the recently published American or European AF guidelines do not support the preferential use of one bleeding score assessment over another.^2,4,5^ It is noted that the previous major scores were developed and validated during a time phrase when AF patients predominantly received vitamin K antagonists (VKAs).^16^ A novel score, termed the DOAC score, was recently developed and validated to enhance the prediction of bleeding risk in patients with AF undergoing treatment with DOACs specifically.^17^ This score showed an enhancement in the predictive capacity for major bleeding events in both the development and derivation cohorts. Nevertheless, the score’s overall performance in predicting major bleeding events remained modest, although it was statistically superior to the HAS-BLED score. Since the DOAC score is a recently developed tool, data are very limited and show inconsistent results regarding its performance in bleeding risk stratification in Asian populations with AF.^18,19^ To fill this gap, we conducted a study comparing how well the new DOAC score works compared with the established HAS-BLED score in assessing bleeding risk among a large retrospective cohort of Asian patients with non-valvular AF receiving DOACs in real-world practice.

## Patients, materials, and methods

### Methods

The study is based in part on data from the Chang Gung Research Database provided by Chang Gung Memorial Hospital (CGMH). The CGMH health system offers the largest and most comprehensive health care services in Taiwan, comprised by a network of 10 hospital branches (three medical centres, five regional/district hospitals, and two municipal hospitals operating under entrustment) located in eight cities.^20^ The hospital network contains a total of 11 360 beds, receives an average of 8.2 million outpatient visits every year, and provides care for at least 2.4 million hospitalized patients annually.^21^ The CGMH system has high overall coverage of Taiwan’s population, with approximately 21.2% for outpatients and 12.4% for inpatients.^22^ The CGMH medical database provides access to each patient’s detailed chart record, diagnosis, laboratory, and imaging data.^23^ Each patient’s identification number are encrypted and de-identified using a consistent encrypting procedure; thus, informed consent was waived in this study. Our present study was approved by the Chang Gung Medical Foundation’s Institutional Review Board (202101936B0C503). The interpretation and conclusions contained herein do not represent the position of CGMH.

### Study cohort

**Figure 1:**
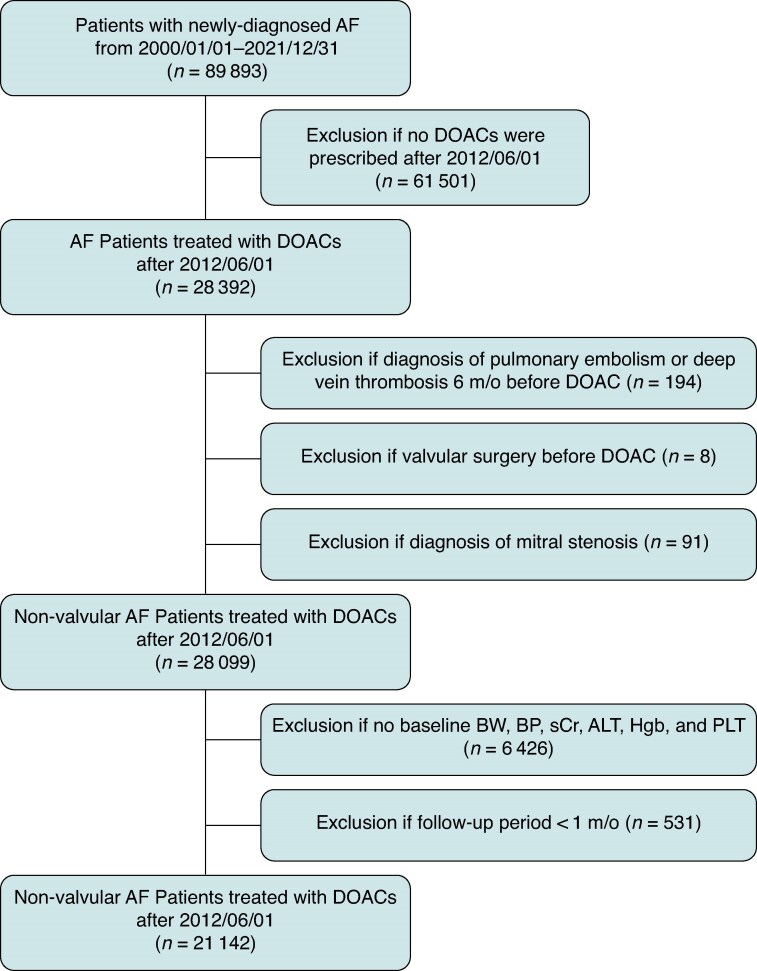
Study design and enrolment of patients with non-valvular AF receiving DOACs. A total of 21 142 patients with non-valvular AF undergoing DOAC therapy, from 1 June 2012 to 31 December 2021, were enrolled in the study. AF, atrial fibrillation; ALT, alanine transaminase; BW, body weight; BP, blood pressure; DOAC, direct oral anticoagulants; Hgb, haemoglobin; sCr, serum creatinine; PLT, platelet.

The flowchart of study design and patient enrolment is shown in *Figure 1*. The CGMH medical database was retrospectively searched for patients who were diagnosed with AF using the ICD-9-CM (427.31) or ICD-10-CM (I48) codes from 1 January 2000 to 31 December 2021 (*n* = 89 893). There were 28 392 patients treated with DOACs after 1 June 2012. Patients with a diagnosis of pulmonary embolism or deep venous thrombosis, post valvular surgery, or mitral stenosis were excluded from the present study. We also excluded those in whom any of the baseline data 12 months within the drug-index date were missing: body weight, serum creatinine (sCr), blood pressure, liver function test, haemoglobin, and platelet count. Patients who had received DOAC therapy for a minimum of 1 month follow-up period were eligible for study inclusion. Finally, a total of 21 142 patients with non-valvular AF treated with DOACs, from 1 June 2012 to 31 December 2021, were enrolled in the present study. The renal function was calculated based on the Cockcroft–Gault formula: [(140 − age) × body weight × 0.85 (if female)]/(72 × sCr).^24^

### Bleeding scores

The HAS-BLED and DOAC scores were calculated for the study cohort of patients according to their original definitions.^11,17^ The methodology for calculating both scores is reported in the Supplementary material online, *Tables S1* and *S2*. The study patients were classified into five categories: (i) very low risk (HAS-BLED = 0 and DOAC score = 0–3), (ii) low risk (HAS-BLED = 1 and DOAC score = 4–5), (iii) intermediate risk (HAS-BLED = 2 and DOAC score = 6–7), (iv) high risk (HAS-BLED 3–4 and DOAC score = 8–9), and (v) very high risk (HAS-BLED ≥ 5 and DOAC score = 10).^17,25^

### Study outcomes

We reported the clinical outcome of major bleeding that occurred within 1 year after DOAC therapy for the study AF patients. To present misclassification, all study outcomes were defined using the first discharge diagnosis. Major bleeding events were defined according to the criteria of International Society on Thrombosis and Haemostasis as clinically overt bleeding which was fatal or associated with any of the following: (i) involvement of a critical anatomical site (intracranial, gastrointestinal, or other critical site) and (ii) a haemoglobin decrease of ≥2 g/dL, or requiring a blood transfusion or hospitalization.^26^ The follow-up period was defined as the time from the drug-index date until the occurrence of study outcome, mortality, or the end date of the study period (31 December 2021), whichever came first.

### Statistical analysis

Continuous variables were expressed as mean and standard deviation (SD) or as median and interquartile range (IQR). Categorical variables were expressed as counts with percentage. We computed the incidence rate (IR) per 100 person-years of major bleeding events based on the five distinct risk categories for the two scores. The cumulative survival based on bleeding score categories was evaluated using the Kaplan–Meier curves, with differences tested using the log-rank test. Cox proportional hazards regression was performed to calculate relative hazard ratios (HRs) with a 95% confidence interval (CI) for major bleeding events and the HAS-BLED and DOAC scores, utilized as both continuous and categorical variables (very high, high, intermediate, low vs. very low risk categories). We employed receiver operating characteristic (ROC) curve (AUC) to evaluate the discriminating performance of the two scores in the prediction of major bleeding. The comparisons between the two AUCs and Harrell C indices were assessed using the DeLong test.^27^ We generated the calibration plots to illustrate the IR of major bleeding events for each score category in our cohort compared to those documented in the original derivation cohort of the two scores.^11,17^ The net reclassification improvement (NRI) index and integrated discrimination index (IDI) were utilized to assess and compare the two scores regarding their ability to reclassify patients.^28^ The NRI index evaluates the efficacy of new models in differentiating between events and non-events. The enhancement in the slopes of the discrimination curve was measured using IDI. Decision curve analysis (DCA) was performed to assess the clinical utility of two scores.^29^ Subgroup analyses were performed to evaluate the model performance for the HAS-BLED and DOAC scores including AUC, NRI, IDI, and DCA stratified by DOAC subtype. A two-sided *P* value of < 0.05 was considered statistically significant. All statistical analyses were performed using SPSS, version 26.0 (IBM Corp., Armonk, NY, USA) and R Statistics, version 4.3.3 (R Foundation for Statistical Computing, Vienna, Austria).

## Results

### Baseline characteristics of patients with non-valvular atrial fibrillation receiving direct oral anticoagulant therapy

A total of 21 142 patients receiving DOAC therapy were eligible for the present study. *Table 1* summarizes the baseline demographic characteristics of the study group. Mean patient age was 75.9 ± 11.0 years and 41% were female. There were 4854 (32%), 7678 (36%), 5252 (25%), and 3358 (16%) patients receiving dabigatran, rivaroxaban, apixaban, and edoxaban, respectively. Major bleeding events occurred in 681 patients in 1-year follow-up (3.66%/year). There were 82 (0.43%/year) intracranial haemorrhages (ICH) occurred for the overall study group. Sixty-one patients were switched from one DOAC to another due to major bleeding events (12, 19, 8, and 22 patients were switched from apixaban, dabigatran, edoxaban, and rivaroxaban, respectively, to another DOAC). Compared with patients without major bleeding events, those with major bleeding events were older and had higher prevalence of stroke or transient ischaemic attack, hypertension, diabetes, heart failure, cancer, and previous history of major bleeding. Patients exhibiting major bleeding have lower haemoglobin and creatinine clearance values. Additionally, a higher proportion of patients with major bleeding events were prescribed proton pump inhibitors, H2 blockers, and diuretics, while a lower proportion were prescribed anti-arrhythmic drugs (*P* < 0.05).

**Table 1:** Characteristics of patients with non-valvular AF receiving DOACs

Baseline characteristics	All patients (*n* = 21 142)	Major bleeding		*P* value
		No (*n* = 20 461)	Yes (*n* = 681)	
Age, yrs	75.9 ± 11.0	75.7 ± 11.0	80.8 ± 9.3	<0.001
Male, *n* (%)	12 417 (59)	12 071 (59)	346 (51)	<0.001
CHA_2_DS_2_-VASc score, median (IQR)	4 (2, 5)	4 (2, 5)	4 (3, 6)	<0.001
CHA_2_DS_2_-VA score, median (IQR)	3 (2, 4)	3 (2, 4)	4 (3, 5)	<0.001
HAS-BLED score, median (IQR)	3 (2, 4)	3 (2, 4)	4 (3, 5)	<0.001
DOAC score, median (IQR)	7 (5, 9)	7 (4, 9)	8 (7, 10)	<0.001
Past medical history, *n* (%)				
Chronic lung disease	1874 (9)	1777 (9)	97 (14)	<0.001
Chronic liver disease	4256 (20)	4097 (20)	159 (23)	0.033
Congestive heart failure	3065 (14)	2913 (14)	152 (22)	<0.001
Hypertension	13 738 (65)	13 228 (65)	510 (75)	<0.001
Hyperlipidaemia	8594 (41)	80 (41)	284 (42)	0.569
Diabetes mellitus	6693 (32)	6432 (31)	261 (38)	<0.001
Stroke	3312 (16)	3154 (15)	158 (23)	<0.001
TIA	482 (2)	454 (2)	28 (4)	0.001
Ischaemic heart disease	6892 (33)	6634 (32)	258 (38)	0.003
Acute coronary syndrome	924 (4)	880 (4)	44 (6)	0.007
Gout	3227 (15)	3082 (15)	145 (21)	<0.001
Peripheral artery disease	1240 (6)	1175 (6)	65 (10)	<0.001
Malignancy	3212 (15)	3068 (15)	144 (21)	<0.001
Major bleeding history	3376 (16)	3187 (16)	189 (28)	<0.001
Baseline laboratory data				
Haemoglobin, g/dL	12.8 ± 1.9	12.9 ± 1.8	11.1 ± 1.6	<0.001
Platelet, ×1000/µL	205.2 ± 59.7	205.3 ± 59.5	202.3 ± 64.0	0.195
CrCl, mL/min	56.3 ± 25.8	56.8 ± 25.9	42.8 ± 21.0	<0.001
ALT, U/L	33.5 ± 48.8	33.5 ± 49.2	32.2 ± 31.6	0.471
Body weight, kg	66.5 ± 13.0	66.5 ± 13.0	67.3 ± 13.5	0.117
Body mass index, kg/m^2^	26.1 ± 4.9	26.1 ± 4.9	26.2 ± 4.5	0.404
SBP, mmHg	134.4 ± 12.9	134.4 ± 12.9	134.2 ± 13.2	0.671
DBP, mmHg	75.0 ± 7.9	75.1 ± 7.9	73.3 ± 7.9	<0.001
HR, bpm	78.3 ± 10.0	78.3 ± 10.0	78.9 ± 9.7	0.096
Baseline medications, *n* (%)				
Use of NSAIDs	3314 (16)	3179 (16)	135 (20)	0.002
Use of PPI	2733 (13)	2588 (13)	145 (21)	<0.001
Use of H_2_ blocker	3894 (18)	3739 (18)	155 (23)	0.003
Use of RAAS inhibitor	11 087 (52)	10 718 (52)	369 (54)	0.354
Use of loop diuretics	4810 (23)	4565 (22)	245 (36)	<0.001
Use of MRA	1714 (8)	1621 (8)	93 (14)	<0.001
Use of thiazide	2898 (14)	2765 (14)	133 (20)	<0.001
Use of amiodarone	3702 (18)	3597 (18)	105 (15)	0.114
Use of dronedarone	694 (3)	679 (3)	15 (2)	0.108
Use of propafenone	1417 (7)	1400 (7)	17 (2)	<0.001
Use of flecainide	486 (2)	479 (2)	7 (1)	0.024
Use of beta-blocker	10 856 (51)	10 512 (51)	344 (51)	0.658
Use of diltiazem	2556 (12)	2462 (12)	94 (14)	0.163
Use of verapamil	783 (4)	764 (4)	19 (3)	0.199
Use of digoxin	2336 (11)	2252 (11)	84 (12)	0.277
Use of statin	6362 (30)	6158 (30)	204 (30)	0.937
Use of azi-/clari-/erythromycin	303 (1)	294 (1)	9 (1)	0.803
Use of anti-fungal agent	246 (1)	233 (1)	13 (2)	0.065
Use of cyclosporin	24 (0)	23 (0)	1 (0)	0.793
Use of antiplatelet agent	9130 (43)	8830 (43)	300 (44)	0.642
Use of DOAC				
Dabigatran	4854 (23)	4723 (23)	131 (19)	0.013
Rivaroxaban	7678 (36)	7413 (36)	265 (39)	0.150
Apixaban	5252 (25)	5066 (25)	186 (27)	0.136
Edoxaban	3358 (16)	3259 (16)	99 (15)	0.312

Data are expressed as the mean ± standard deviation (SD) (med, IQR), or as percentage.

AF, atrial fibrillation; ALT, alanine aminotransferase; CrCl, creatinine clearance; DBP, diastolic blood pressure; DOAC, direct oral anticoagulant; HR, heart rate; IQR, interquartile range; MRA, mineralocorticoid receptor antagonist; NSAIDs, non-steroidal anti-inflammatory drugs; PPI, proton pump inhibitor; RAAS, renin-angiotensin-aldosterone system; SBP, systolic blood pressure; TIA, transient ischaemic attack.

### Risk of major bleeding according to the HAS-BLED and DOAC scores

**Figure 2:**
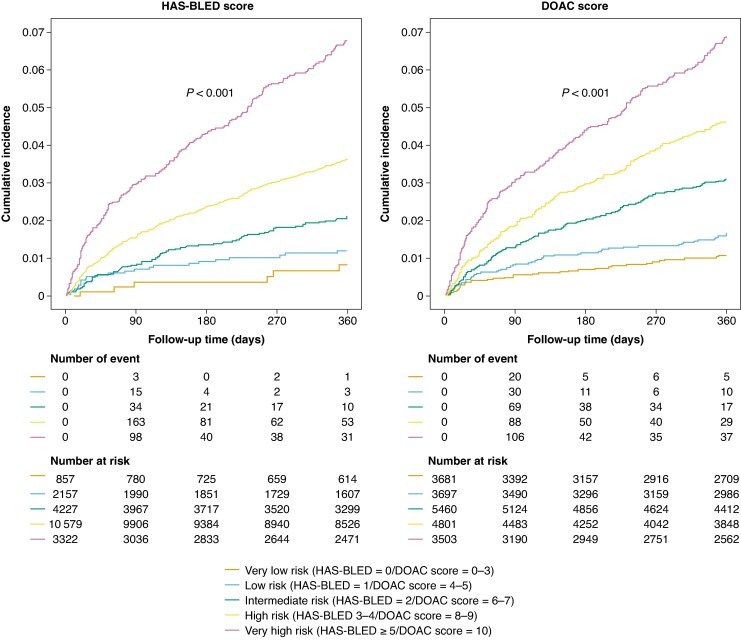
Cumulative incidence for major bleeding events over time with patients classified using the DOAC and HAS-BLED scores by predicted risk category. The cumulative incidence of major bleeding events categorized by the HAS-BLED and DOAC scores. Patients categorized as different bleeding risk according to both the HAS-BLED and DOAC scores showed an increment in the cumulative risk of major bleeding events. The study patients were classified into five categories: (i) very low risk (HAS-BLED = 0 and DOAC score = 0–3), (ii) low risk (HAS-BLED = 1 and DOAC score = 4–5), (iii) intermediate risk (HAS-BLED = 2 and DOAC score = 6–7), (iv) high risk (HAS-BLED 3–4 and DOAC score = 8–9), and (v) very high risk (HAS-BLED ≥ 5 and DOAC score = 10). The abbreviations as in *Figure 1*.

According to the HAS-BLED score, 857 (4.1%) and 2157 (10.2%) patients were considered at very low and low risks, while 10 579 (50.0%) and 3322 (15.7%) patients were at high and very high risks. Using the DOAC score, 3681 (17.4%) and 3697 (17.5%) patients were categorized as very low and low risks, while 4801 (22.7%) and 3503 (16.6%) were at high and very high risks. Patients categorized as different bleeding risks according to both the HAS-BLED and DOAC scores showed an increment in the cumulative risk of major bleeding events (*Figure 2*). Consistently, the risk of major bleeding progressively and significantly increased according to the different categories defined by the DOAC score compared to the very low-risk group using the Cox regression analysis (*Table 2*). Of note, the low-risk category was significantly associated with major bleeding compared to the very low-risk category (HR 1.53, 95% CI 1.01–2.32) for the DOAC score, but this association was not significant for the HAS-BLED score. After multivariate adjustment of all covariates in *Table 1* except for the components of HAS-BLED and DOAC scores, this association of low-risk category with major bleeding was not significant either for the HAS-BLED or DOAC score. Patients considered as intermediate, high-risk, and very-high risk according to the HAS-BLED and DOAC scores both showed a significant increase in the risk of major bleeding (*Table 2*).

**Table 2:** Cox regression analysis for major bleeding according to risk categories for the HAS-BLED and DOAC scores

	*N*	One-year rate (%)	Univariable		Multivariate	
			HR [95% CI]	*P* value	aHR^a^ [95% CI]	*P* value
HAS-BLED score						
Continuous			1.43 [1.36–1.51]	<0.001	1.36 [1.28–1.45]	<0.001
≥3 (vs. <3)			2.63 [2.15–3.22]	<0.001	2.25 [1.81–2.80]	<0.001
Very low (0)	857	0.83	Ref.		Ref.	
Low (1)	2157	1.29	1.57 [0.64–3.84]	0.324	1.59 [0.65–3.88]	0.313
Intermediate (2)	4227	2.19	2.29 [1.17–3.62]	0.019	2.62 [1.14–6.01]	0.023
High (3–4)	10 579	3.82	4.70 [2.10–10.53]	<0.001	4.40 [1.95–9.91]	<0.001
Very high (≥5)	3322	7.30	8.90 [3.95–20.03]	<0.001	7.18 [3.15–16.39]	<0.001
DOAC score						
Continuous			1.28 [1.23–1.32]	<0.001	1.24 [1.19–1.28]	<0.001
≥8 (vs. <8)			2.66 [2.28–3.11]	<0.001	2.20 [1.85–2.61]	<0.001
Very low (0–3)	3681	1.14	Ref.		Ref.	
Low (4–5)	3697	1.72	1.53 [1.01–2.32]	0.046	1.51 [0.99–2.29]	0.055
Intermediate (6–7)	5460	3.26	2.90 [2.02–4.16]	<0.001	2.71 [1.88–3.90]	<0.001
High (8–9)	4801	4.87	4.33 [3.04–6.17]	<0.001	3.84 [2.67–5.53]	<0.001
Very high (=10)	3503	7.43	6.53 [4.59–9.29]	<0.001	5.11 [3.52–7.43]	<0.001

The study patients were classified into five categories: very low risk (HAS-BLED = 0 and DOAC score = 0–3), low risk (HAS-BLED = 1 and DOAC score = 4–5), intermediate risk (HAS-BLED = 2 and DOAC score = 6–7), high risk (HAS-BLED 3–4 and DOAC score = 8–9), and very high risk (HAS-BLED ≥ 5 and DOAC score = 10).

aHR, adjusted hazard ratio, CI, confidence interval; HR, hazard ratio.

^a^All covariates in *Table 1*, except for the components of HAS-BLED and DOAC scores, were included in the adjustment.

**Figure 3:**
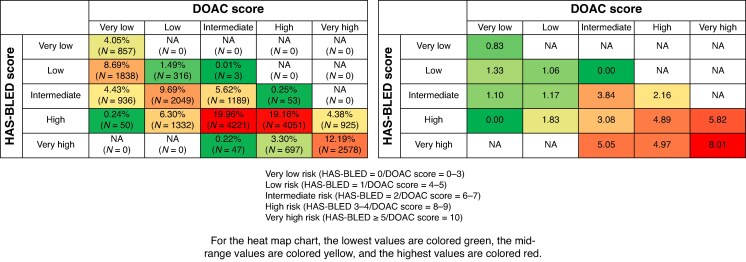
The population proportion and cumulative incidence of major bleeding events categorized by the HAS-BLED and DOAC scores together. Among patients classified as ‘high risk’ by the HAS-BLED score, a notable proportion were actually categorized by the DOAC score into very low (0.24%), low (6.30%), and intermediate (19.96%) risk groups. The annual incidence of major bleeding in these three DOAC score groups was 0%, 1.83%, and 3.08%, respectively, which were lower than the rates observed in patients classified as high (4.89%) and very high (5.82%) risk by the DOAC score within the same HAS-BLED category. The abbreviations as in *Figure 1*.

Among patients classified as ‘high risk’ by the HAS-BLED score, a notable proportion were actually categorized by the DOAC score into very low (0.24%), low (6.30%), and intermediate (19.96%) risk groups. The annual incidence of major bleeding in these three DOAC score groups was 0%, 1.83%, and 3.08%, respectively, which were lower than the rates observed in patients classified as high (4.89%) and very high (5.82%) risk by the DOAC score within the same HAS-BLED category (*Figure 3*).

**Figure 4:**
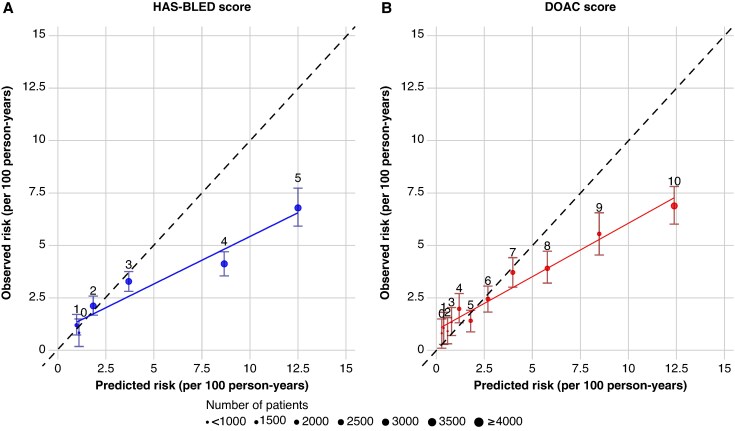
Calibration curves for bleeding risk scores plotted against original derivation cohorts for the HAS-BLED and DOAC score. Both the HAS-BLED and DOAC scores showed a good calibration for the very-low, low, to intermediate risk categories (HAS-BLED < 3 and DOAC score < 8), while the two bleeding risk scores both overestimate the major bleeding risk when comparing the predicted risk with the observed annualized event rate for the high and very-high risk categories (HAS-BLED ≥ 3 and DOAC score ≥ 8). The abbreviations as in *Figure 1*.

For the model calibration analysis comparing the IR of major bleeding events in our present cohort with those reported in the original derivation cohorts,^11,17^ both the HAS-BLED and DOAC scores showed a good calibration for the very-low, low, to intermediate-risk categories, while the two bleeding risk scores both overestimate the major bleeding risk when comparing the predicted risk with the observed annualized event rate for the high- and very-high-risk categories (HAS-BLED ≥ 3 and DOAC score ≥ 8) (*Figure 4*).

### Predictive performance and reclassification analysis

**Figure 5:**
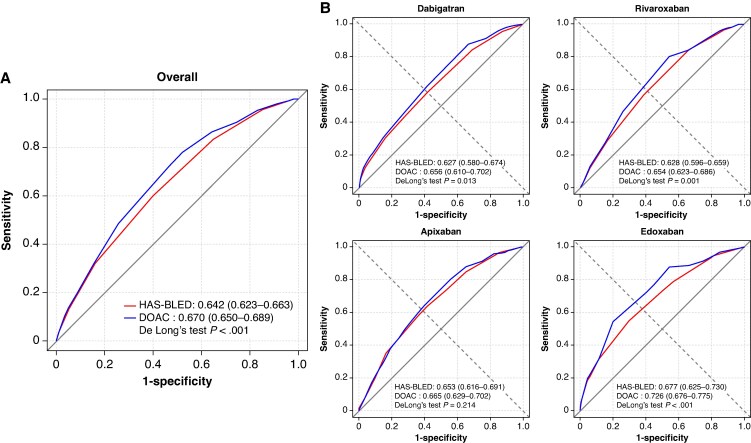
Area under the curve for prediction of major bleeding events for HAS-BLED and DOAC scores (*A*) and categorized by different DOACs (*B*). The DOAC score had a significantly higher AUC value as compared with the HAS-BLED score. Further analysis showed a consistent difference for the dabigatran, rivaroxaban, and edoxaban, but not for the apixaban. AUC, area under the curve; CI, confidence interval. Other abbreviations as in *Figure 1*.

*Figure 5* shows the ROC curves for the HAS-BLED and DOAC scores. Both scores had a moderate performance in the prediction of major bleeding events. The DOAC score had a significantly higher AUC value [AUC 0.670 (95% CI 0.650–0.689)] when compared to the HAS-BLED score [AUC 0.642 (95% CI 0.623–0.663)] using the DeLong test (*P* for difference < 0.001). We conducted a sensitivity analysis restricting our cohort to patients with ≥12 months of continuous DOAC exposure. There were 15 238 patients (72.1%) patients with ≥12 months of continuous DOAC exposure. The result remained consistent with our primary analysis [0.663 (0.637–0.688) vs. 0.629 (0.602–0.655) for DOAC score vs. HAS-CLED score; *P* < 0.001] (see Supplementary material online, *Figure S1*). Also, the sensitivity analysis conducted on the population receiving different DOAC regimens showed a consistent difference between the ROC curves regarding major bleeding for dabigatran [0.656 (0.610–0.702) vs. 0.627 (0.580–0.674); *P* = 0.013], rivaroxaban [0.654 (0.623–0.686) vs. 0.628 (0.596–0.659); *P* = 0.001], and edoxaban [0.726 (0.676–0.776) vs. 0.677 (0.625–0.730); *P* < 0.001], except for the apixaban, which showed no difference between the ROC curves [0.665 (0.629–0.702) vs. 0.653 (0.616–0.691); *P* = 0.214].

We analyse the performance of the two scores stratified by different age subgroups. There were 39 of 2961 (1.32%), 125 of 5944 (2.10%), 251 of 7361 (3.41%), and 266 of 4876 (5.46%) major bleeding events in patients with age <65, 65–74, 75–84, and ≥85 years of age, respectively. Subgroup analyses revealed that the superior predictive performance of the DOAC score compared to the HAS-BLED score was primarily observed in patients aged 75–84 years of age [0.615 (0.582–0.648) vs. 0.580 (0.545–0.615); *P* < 0.001] and ≥85 years of age [0.600 (0.564–0.637) vs. 0.566 (0.529–0.604); *P* < 0.001] (see Supplementary material online, *Figure S2*). We further analyse the performance of the two scores in the prediction of different bleeding types. The DOAC score had a significantly higher AUC value than the HAS-BLED score in the prediction of major gastrointestinal bleeding [0.688 (0.660–0.716) vs. 0.648 (0.618–0.679); *P* < 0.001] rather than ICH [0.561 (0.497–0.624) vs. 0.576 (0.515–0.637); *P* = 0.366] (see Supplementary material online, *Figure S3*).

**Figure 6:**
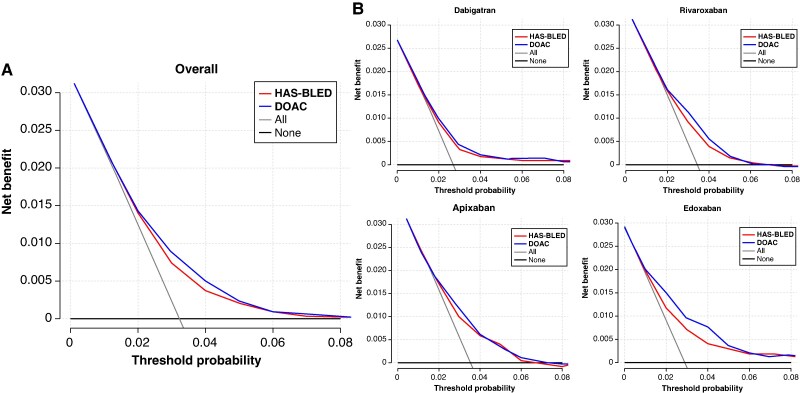
DCA showing the clinical efficacy for the DOAC and HAS-BLED scores (*A*) and categorized by different DOACs (*B*). The DCA showed that the DOAC score outperforms the HAS-BLED score in terms of net benefit across the bleeding risk threshold in the range of around 2–5% (*A*). It is noted that the clinical utility of the two scores for apixaban was not different since the curve of the net benefit of the models overlapped across different risk thresholds (*B*). DCA, decision curve analysis. Other abbreviations as in *Figure 1*.

*Table 3* shows the results of reclassification analyses. The DAOC score showed an improvement in terms of NRI, IDI, and MI index for 1-year major bleeding compared with the HAS-BLED score. Also, the sensitivity analysis showed a consistent improvement for dabigatran, rivaroxaban, and edoxaban, but not for apixaban. The DCA showed that the DOAC score outperforms the HAS-BLED score in terms of net benefit across the bleeding risk threshold in the range of around 2–5% (*Figure 6A*). It is noted that the clinical utility of the two scores for apixaban was not different since the curve of the net benefit of the models overlapped across different risk thresholds (*Figure 6B*).

**Table 3:** Reclassification analysis for DOAC score vs. HAS-BLED scores for assessment of major bleeding risk

DOAC score vs. HAS-BLED score						
Comparison	NRI (95% CI) at 1 year	*P* value	IDI (95% CI) at 1 year	*P* value	MI (95% CI) at 1 year	*P* value
Overall	25.7% (18.1–33.2%)	<0.001	0.26% (0.17–0.35%)	<0.001	0.36% (0.36–0.41%)	<0.001
Dabigatran	35.3% (18.3–52.2%)	<0.001	0.34% (0.14–0.54%)	<0.001	0.31% (0.31–0.58%)	<0.001
Rivaroxaban	31.2% (19.4–43.1%)	<0.001	0.25% (0.12–0.39%)	<0.001	0.40% (0.34–0.59%)	<0.001
Apixaban	11.7% (−27.9–26.4%)	0.115	0.03% (−0.12–0.20%)	0.683	0.31% (−0.00–0.31%)	0.184
Edoxaban	36.5% (16.4–56.1%)	<0.001	0.58% (0.24–0.93%)	0.001	0.75% (0.23–0.78%)	0.008

CI, confidence interval; IDI, integrated discrimination improvement; MI, median improvement; NRI, net reclassification index.

## Discussion

To the best of our knowledge, this is the largest study to validate the DOAC risk score for major bleeding assessment, with a focus on Asian patients with non-valvular AF receiving DOAC therapy. The main findings of this study are as follows: (i) the DOAC score better identified patients at high risk of bleeding when compared to the HAS-BLED score; (ii) both the DOAC scores and HAS-BLED scores are associated with a significant risk of major bleeding events, with only modest predictive performance (AUC < 0.7); (iii) the DOAC score had a significantly higher AUC value compared with the HAS-BLED score. Further analysis showed a consistent difference between the ROAC curves for dabigatran, rivaroxaban, and edoxaban, but not for apixaban. Subgroup analyses indicated that the superiority of the DOAC score over the HAS-BLED score is primarily driven by elderly patients (≥75 years) and risk prediction in gastrointestinal bleeding; (iv) reclassification analyses showed a significant difference between the two scores across the bleeding risk threshold in the range of 2–5%, favouring the DOAC score; and (v) both the two scores showed a good calibration for the very-low, low, and intermediate risk categories, while the two bleeding risk scores both overestimate the risk of major bleeding risk when comparing the predicted risk with the observed risk for the high and very-high risk categories (HAS-BLED ≥ 3 and DOAC score ≥ 8). We propose that the DOAC score may be a valuable tool for bleeding risk assessment in Asian patients with non-valvular AF receiving DOAC therapy, particularly those who are at low to intermediate risk for bleeding.

The higher predictive performance of the DOAC score compared with the HAS-BLED score is primarily attributed to its more detailed assessment of risk factors.^17^ For example, age was subdivided into substrata in DOAC score (e.g. 2 points for age 65–69 and 5 points for age ≥ 80 years of age), enabling a more accurate assessment of individual risk than the generalized age categories (e.g. age ≥ 65 years of age or not) used in HAS-BLED. For kidney dysfunction, a critical bleeding risk factor and dosage-adjustment consideration in patients receiving DOAC therapy, the DOAC score was subdivided into three subcategories (e.g. creatinine clearance 30–60 mL/min scores 1 point and <30 mL/min scores 2 points), which reflects risk more accurately than the binary classification used in the HAS-BLED score. The use of antiplatelet treatment in the DOAC score was further modelled into subcategories with monotherapy for 2 points and dual therapy for 3 points, while the use of nonsteroidal anti-inflammatory drugs (NSAIDs) was recognized as an independent risk factor (1 point). The incorporation of low body weight (body mass index < 18.5 kg/m²) as a separate risk factor (1 point) may improve predictive accuracy, given that underweight associated with an increased risk of major bleeding, a factor not considered in the HAS-BLED score.^30^

Until now, limited real-word studies comparing the DOAC score and the HAS-BLED score for bleeding risk assessment in AF patients receiving OACs have yielded inconsistent results. One Spanish retrospective registry study examined a total of 14 672 AF patients receiving DOAC (10.1%), VKAs (66.3%), or non-OAC therapy (23.6%). In patients receiving DOAC therapy (*n* = 1484), the DOAC score (C-statistic = 0.711) significantly outperformed HAS-BLED (*C* = 0.640, *P* = 0.03). Decision curve analysis indicated that the DOAC score provided better net benefit across all bleeding risk thresholds, consistently outperforming HAS-BLED with fewer false positives without increasing false negatives.^31^ Another retrospective multicentre cohort study examined the association between the DOAC score and bleeding events in patients with AF undergoing transcatheter aortic valve replacement (TAVR) utilized data from the Japanese multicentre registry (OCEAN-TAVI). A total of 1230 patients (mean age 84.6 ± 5.1 years) were ultimately included in the analysis. Time-dependent ROC analysis showed that the DOAC score exhibited significantly higher predictive accuracy for bleeding events than the HAS-BLED score in the overall cohort (AUC 0.592 vs. 0.509, adjusted *P* = 0.04). The study concludes that the DOAC score is significantly associated with bleeding events in AF patients after TAVR.^19^ However, other studies have shown comparable performance between the two scoring systems. A European prospective observational registry including 2834 AF patients treated with DOACs reported only modest predictive power for both scores, with AUC values of 0.65 for HAS-BLED and 0.62 for DOAC (*P* = 0.332). Furthermore, a poor calibration for the DOAC score was observed especially in high-risk patients. Also, no significant difference in NRI was noted for DOAC score compared to HAS-BLED score.^25^ Another retrospective real-world study compared the two scores in Chinese patients with non-valvular AF receiving DOACs (*n* = 2532), showing no significant difference in predicting major bleeding events for DOAC scores compared to HAS-BLED scores. The calibration performance of the HAS-BLED score was superior to DOAC score.^18^

The annualized IRs of major bleeding and ICH events were 3.66% and 0.43%/year in our multicentre hospital-based cohort of AF patients receiving DOAC therapy. Consistently, the annualized event rate of major bleeding (ICH) events reported in the pivotal NOAC trials was 2.17 (0.45), 3.44 (0.59), 2.02 (0.67), and 2.86 (0.60) %/year for the Asian population receiving dabigatran, rivaroxaban, apixaban, and edoxaban, respectively.^32–35^ Compared with our study, a compatible rate of major bleeding stratified by the predicted risk category was reported in the GARFIELD-AF and RAMQ registries.^17^ Of note, the incidence of observed major bleeding was notably lower in the present cohort than the predicted bleeding rates derived from the original HAS-BLED and DOAC scores, specifically in the high- and very-high-risk categories (HAS-BLED ≥ 3 and DOAC score ≥ 8).^11,15,17^ These findings align with a recent study reporting identical patterns in one prospective real-world cohort in Europe, with both HAS-BLED and DOAC scores showing poorer calibration at high-risk values.^25^ This consistent overestimation likely reflects enhanced clinical vigilance for high-risk patients, evolution of anticoagulation management since score development, and population differences from original derivation cohorts. Nevertheless, this discrepancy may be attributed to several factors, including a higher sensitivity in ascertaining bleeding outcomes in the prospective randomized pivotal trials used for the original score derivation compared with observational cohorts. Furthermore, the HAS-BLED score was developed during the warfarin era and may overestimate the bleeding risk in patients receiving DOACs with more favourable safety profiles. Several global registries have shown that 15–25% of patients on DOACs, especially in the Asian population, may be receiving doses below guideline-recommended levels in real-world practice.^36–38^ The real-world database may also report a lower bleeding rate because of the limitations of claiming code outcome ascertainment. These findings highlight the need for score recalibration in contemporary populations.

The advantage of our study cohort in validating the performance of two bleeding scores lies in the fact that all patients in our present study were exclusively receiving DOACs (*n* = 21 142). This is a critical distinction because previous study that assessed the predictive performance of these scores included mixed populations of anticoagulated patients, with a substantial proportion receiving VKAs rather than DOACs.^31^ The inclusion of VKA users in these studies introduces a significant source of variability because the bleeding risk associated with VKAs is influenced by factors such as labile INR, dietary interactions, and frequent monitoring, which are not relevant to DOAC therapy. Another strength of the present study is that we also directly compare the performance of two scores across four DOACs (dabigatran, *n* = 4854, rivaroxaban, *n* = 7678; apixaban, *n* = 5252; edoxaban, *n* = 3358), showing heterogeneous results across these agents. There was no difference in the ROC curve between the two scores for patients receiving apixaban. Apixaban is often preferentially prescribed to older, impaired renal function, or higher-risk patients precisely because of its safer bleeding profile in pivotal trial and real-world practice. This might lead to a more homogeneous high-risk population when apixaban is used, making it harder for any risk score to achieve high discriminatory power. Conversely, the edoxaban subgroup showed the largest difference in predictive performance between the HAS-BLED and DOAC scores. The edoxaban subgroup was smaller than other DOAC subgroups, which could lead to greater statistical variability and more pronounced differences. The prescribing patterns of edoxaban might differ from other DOACs, potentially enrolling certain high-risk patient characteristics in this subgroup. Furthermore, edoxaban has distinct pharmacokinetic properties and dosing considerations (50% and 50% of kidney and hepatic elimination, respectively) that may interact differently with the risk factors captured by each score.^39^ Edoxaban was introduced later in the study period, as this could reflect evolving prescribing practices or patient populations. Previous studies did not focus on a specific DOAC type, possibly due to a limited patient number receiving DOAC therapy overall, leading to a limited understanding of the predictive accuracy of these bleeding scores in patients exclusively treated with different DOACs. Our present study indicates a superior predictive performance of the DOAC score compared with the HAS-BLED score with heterogeneity of different DOACs, which requires further validation in different DOAC cohorts to determine whether this represents a reproducible drug-specific phenomenon or a study-specific observation.

The superior predictive performance of the DOAC score compared to the HAS-BLED score showed both age-dependent and bleeding type-specific patterns that reflect important clinical and methodological considerations. The superiority of the DOAC score in patients ≥ 75 years suggests mechanistic relevance, as older patients may have distinct bleeding risk profiles that the DOAC score captures more effectively, while also reflecting a design advantage given that the DOAC score was developed specifically for DOAC patients who tend to be older than VKA patients in many cohorts,^11,17^ indicating that risk stratification strategies might benefit from age-specific approaches. Similarly, the better performance in gastrointestinal bleeding compared to ICH prediction reflects the DOAC score's anatomical specificity, as it was designed with particular attention to gastrointestinal bleeding mechanisms, with risk factor weighting that may be more relevant to gastrointestinal bleeding pathophysiology, and represents the clinical context where gastrointestinal bleeding is more common in patients receiving DOAC therapy and constitutes the majority of bleeding events in this population.

Although the AUC between the DOAC score and HAS-BLED score was significant, the absolute difference of 0.028 is only modest. In addition, the DCA revealed only modest differences for the two scores, with maximal net benefit differences of 0.10–0.15% in terms of net benefit with the bleeding risk threshold in the range of 2–5%, suggesting that the clinical advantage of the DOAC score over the HAS-BLED score is marginal. These findings suggest that while the DOAC score represents a statistical improvement over the HAS-BLED score, the practical clinical benefit may not justify widespread replacement of the HAS-BLED score in all clinical settings. Furthermore, the superior discriminative ability and more granular risk stratification of the DOAC score over the HAS-BLED score are justified by the additional complexity requiring assessment of more variables than the HAS-BLED score. Clinical practice should judge the trade-off between usability and accuracy individually. The primary aim of our comparison was to evaluate relative performance rather than to advocate for the replacement of one score with the other. The major AF guidelines do not recommend one specific bleeding score as superior to others and have emphasized that bleeding risk assessment should not be used to withhold anticoagulation in patients who would otherwise benefit from stroke prevention.^4,5^ However, such a small improvement in discrimination ability can be relevant in large populations, particularly when the bleeding scores can be used to guide preventive strategies or stratify bleeding risk for quality-improvement purposes.

While the present study was performed exclusively in an Asian population, several reasons support the potential broader applicability of our findings. Previous studies indicated that Asian patients had higher plasma concentrations of DOACs compared to non-Asian patients when given the same dose.^40,41^ These differences are attributed to genetic polymorphisms in drug-metabolizing enzymes (particularly CYP3A4) and transporters (like P-glycoprotein), lower body weight, and differences in drug distribution.^42–44^ Despite Asians taking warfarin having a higher risk of major bleeding when compared to non-Asians in the NOAC pivotal trials in AF (3.82% vs. 3.53%, 5.14% vs. 3.35%, 3.84% vs. 3.00%, and 4.80% vs. 3.29% for the RE-LY, ROCKET-AF, ARITOTOLE, and ENGAE AF-TIMI 48 trials), the absolute risk of major bleeding with NOACs was consistent between Asian and non-Asian patients in the pivotal trials, with annual major bleeding risks of 2.22% vs. 2.99%, 3.44% vs. 3.61%, 2.02% vs. 2.02%, and 2.15% vs. 2.86% for the dabigatran (110 mg), rivaroxaban (20/15 mg), apixaban (5/2.5 mg), and edoxaban (60/30 mg) respectively,^32–35^ showing comparable bleeding risk profiles across Asian and non-Asian groups. Also, both the HAS-BLED and DOAC scores have been validated and show consistent performance in Asian cohorts,^18,19^ further supporting the potential generalizability of our results to non-Asian populations.

### Study limitations

This study has several limitations. The HAS-BLED is typically dichotomized (≥3 vs. <3) and the DOAC score is categorized into five groups (very low, low, moderate, high, and very high).^11,17^ In our present analysis, we applied a five-tier classification to both scores to allow finer granularity in bleeding risk assessment, enable direct comparison between the two scores on a uniform scale, and explore potential risk heterogeneity within the conventional categories. This approach was intended for exploratory purposes and is not proposed as a change to standard clinical use. Nevertheless, different arbitrary categorizations may cause different results and limit interpretability. The renal function used in the DOAC score was defined according to the Cockcroft–Gault formula. Applying alternative equations, such as MDRD or CKD-EPI, may produce different estimates and potentially alter risk stratification.^45^ Due to its retrospective and observational design, the baseline characteristics and medications of each patient may differ depending on their bleeding risk levels in real-world settings. We did not consider the potential impact of time-varying changes in each patient’s laboratory data, medical diagnoses, or prescribed medications on the clinical outcomes observed during the study period. Furthermore, the CGMH database lacked important information regarding lifestyle factors such as smoking, alcohol, and physical activity, which could have influenced our results. The exclusion of labial INR for the HAS-BLED score calculation is another disadvantage. The frailty status has significant impacts on both bleeding and thromboembolic risk stratification for AF management settings, as well as treatment tolerance and outcomes. Frail patients often present with complex risk-benefit profiles that require individualized anticoagulation decisions beyond traditional risk scores.^46,47^ An inherent limitation of our study methodology is the inability to assess the frailty of each patient in a retrospective study. Furthermore, the variables used for the risk score calculation have not been validated in this CGRD database population. The DOAC-dosing information was not available in our dataset, preventing assessment of dose-related bleeding risk variations. The present study was conducted within a single healthcare system, which may limit generalizability to other populations and healthcare settings. We are unable to capture major bleeding events outside of the CGMH system due to a lack of external data connections, which may result in an underestimation of major bleeding events in our study cohort. Due to a lack of data regarding certain variables necessary for the DOAC score calculation, a total of 6426 patients were excluded from the present analysis, which may limit the interpretation of our study’s findings in terms of extrapolation to patient populations with low bleeding risk broadly. The superiority of the DOAC score in specific subgroups (elderly patients and gastrointestinal bleeding) suggests that the overall performance advantage may not apply uniformly across all patient populations and bleeding types. Finally, the study was conducted exclusively on Asians, which may limit the generalizability of the findings to non-Asians, who may have different genetic predispositions and environmental exposures that influence outcomes.

## Conclusions

The DOAC score provides a valuable, tailored bleeding risk assessment tool for Asian patients with non-valvular AF receiving DOAC therapy, thereby enhancing clinical decision-making and facilitating shared discussions between clinicians and patients. The superiority of the DOAC score over the HAS-BLED score is modest and is majorly driven by elderly patients (≥75 years) and the prediction of the risk of gastrointestinal bleeding.

## Supplementary Material

euaf251_Supplementary_Data

## Data Availability

The datasets used in this study are only available in the Chang Gung Medical Data Center, Taiwan. The programs (codes) used in this study are available from the corresponding author on reasonable request.
