# Supplementary material for: Performance of DOAC and HAS-BLED scores in predicting major bleeding in Asian patients with non-valvular atrial fibrillation receiving direct oral anticoagulants
Source: Europace. 2025 Oct 3;27(11):euaf251. doi: 10.1093/europace/euaf251 (PMC12578370; doi:10.1093/europace/euaf251)

Supplemental Table I. Assessment of the HAS-BLED score

Risk Factor	Criteria	Points
**H** Hypertension	Uncontrolled, SBP > 160 mmHg	1
**A** Abnormal renal/liver function	Renal: dialysis, transplant, or sCr > 2.26 mg/dL<br>Liver: cirrhosis, bilirubin > 2× normal or AST/ALT > 3× normal	1<br>+1
**S** Stroke history	Any prior stroke	1
**B** Bleeding	Prior major bleeding history	1
**L** Labile INR	Unstable/high INRs, time in therapeutic range < 60%	1
**E** Elderly	Age > 65	1
**D** Drugs or alcohol	Drugs: aspirin, P2Y12 inhibitors, NSAIDs<br>Alcohol: >8 drinks/week	1<br>+1
**Total score range**		0–9

AST, aspartate transaminase; ALT, alanine aminotransferase; INR, international normalized ratio; NSAIDs, non-steroidal anti-inflammatory drugs; SBP, systolic blood pressure; sCr, serum creatinine

Supplemental Table II. Assessment of the DOAC score

Parameter	Criteria	Points
**Age (years)**	65–69<br>70–74<br>75–79<br>≥80	2<br>3<br>4<br>5
**CrCl (ml/min)**	30–60<br><30	1<br>2
**Underweight**	BMI <18.5 kg/m²	1
**Stroke/TIA history**	Any history	1
**Diabetes**	Present	1
**Hypertension**	Present	1
**Antiplatelet use**	Aspirin	2
	Dual-antiplatelet therapy	3
**NSAID use**	Present	1
**Bleeding history**	Present	3
**Liver disease***	Present	2
**Total score range**	Max score = 10 (scores ≥10 are assigned as 10)	0–10

*Defined as AST, ALT ≥3X upper limit of normal, ALP ≥2X upper limit of normal, or cirrhosis.

BMI, body mass index; CrCl, creatinine clearance; DOAC, direct oral anticoagulant; NSAIDs, non-steroidal anti-inflammatory drugs; TIA, transient ischemic attack

Supplemental Figure I. The sensitivity analysis restricting our cohort to patients with ≥ 12 months of continuous DOAC exposure

There were 15,238 patients (72.1%) patients with ≥ 12 months of continuous DOAC exposure. The result remained consistent with our primary analysis (0.663; [0.637-0.688] vs. 0.629; [0.602-0.615] for DOAC score vs. HAS-CLED score; *P* < .001).

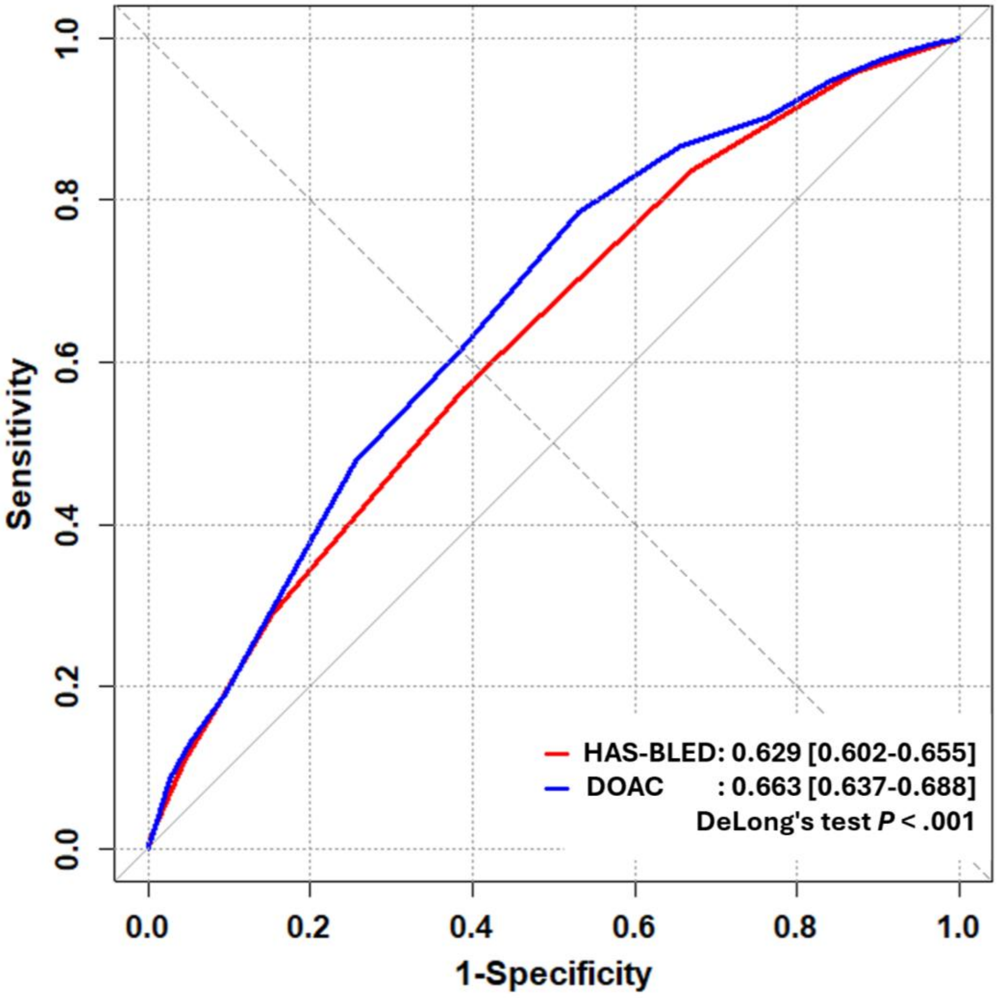

Supplemental Figure II. The subgroup analysis for the performance of the two scores in different age subgroups.

There were 39/2961 (1.32%), 125/5944 (2.10%), 251/7361 (3.41%), and 266/4876 (5.46%) of major bleeding events in patients with age < 65, 65 to 74, 75 to 84, and ≥ 85 years of age, respectively. Subgroup analyses revealed that the superior predictive performance of the DOAC score compared to HAS-BLED was primarily observed in patients aged 75 to 84 years of age (0.615; [0.582-0.648] vs. 0.580; [0.545-0.615]; *P* < .001) and ≥ 85 years of age (0.600; [0.564-0.637] vs. 0.566; [0.529-0.604]; *P* < .001).

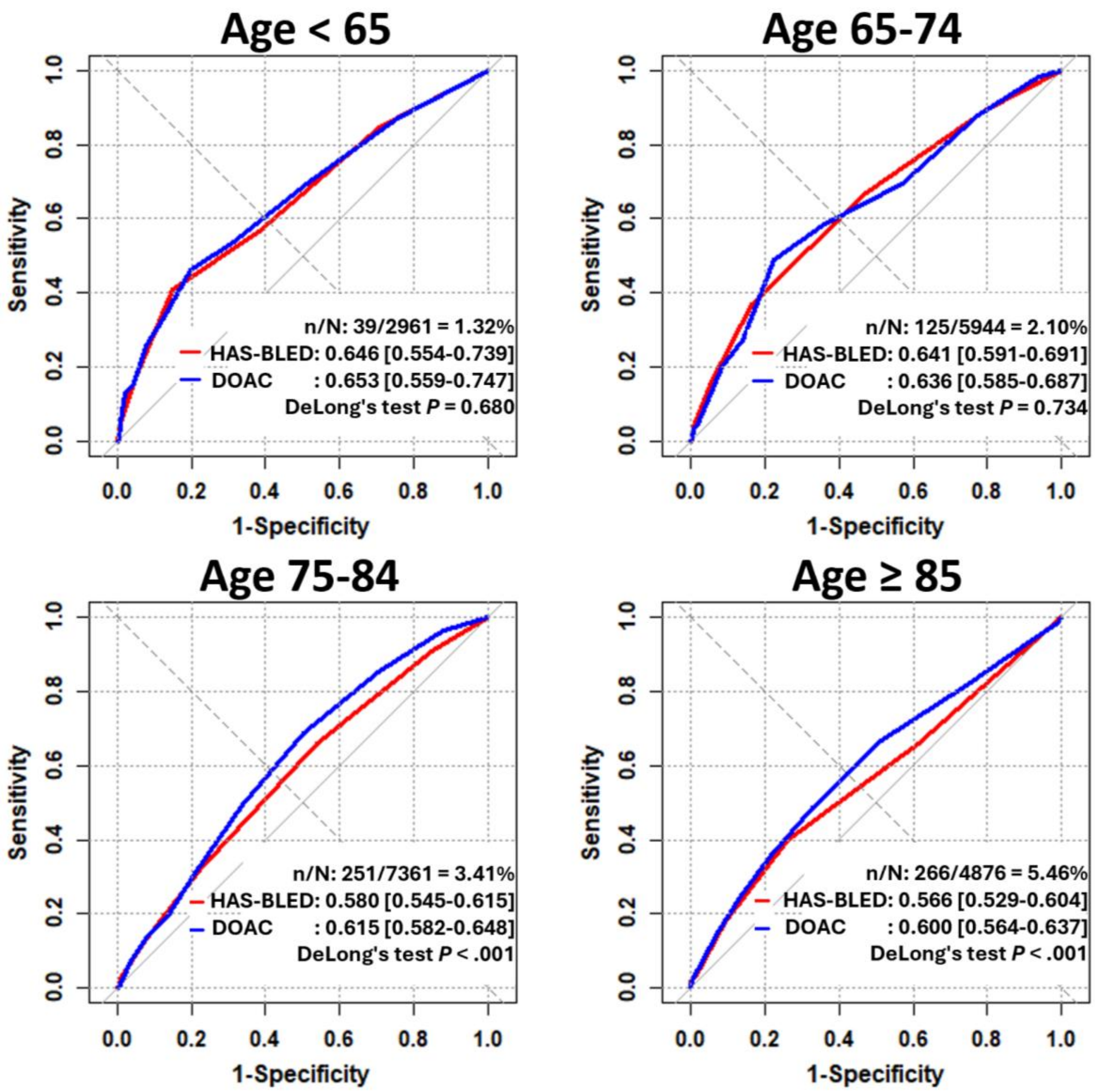

Supplemental Figure III. The subgroup analysis for the performance of the two scores in the prediction of different bleeding types

The DOAC score had a significant higher AUC value than the HAS-BLED score in the prediction of major gastrointestinal bleeding (0.688; [0.660-0.716] vs. 0.648; [0.618-0.679]; *P* < .001) rather than ICH (0.561; [0.497-0.624] vs. 0.576; [0.515-0.637]; *P* = 0.366)

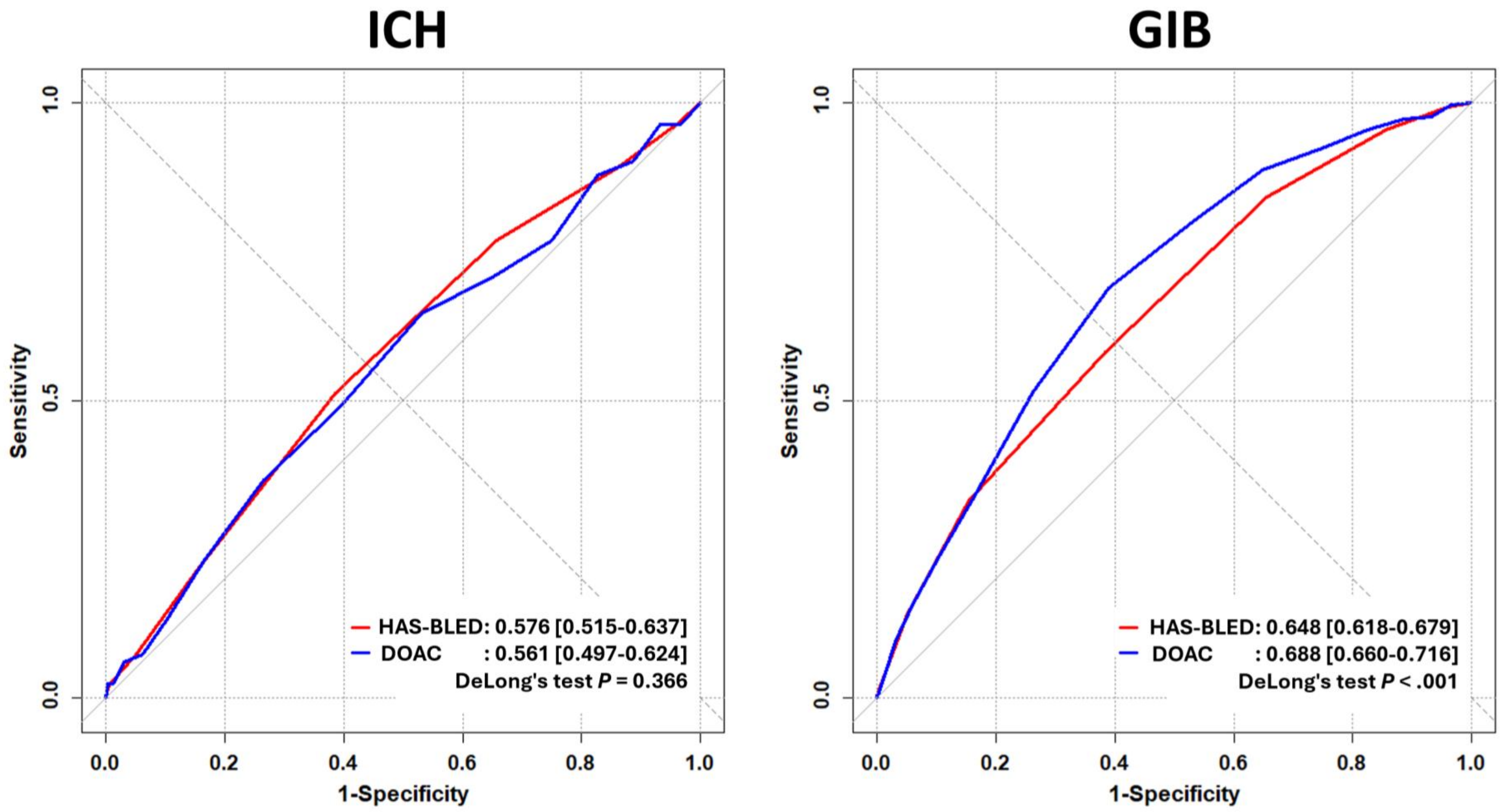

Supplement: euaf251_Supplementary_Data [file euaf251_supplementary_data.pdf]
